# Improving imputation in disease-relevant regions: lessons from cystic fibrosis

**DOI:** 10.1038/s41525-018-0047-6

**Published:** 2018-03-20

**Authors:** Naim Panjwani, Bowei Xiao, Lizhen Xu, Jiafen Gong, Katherine Keenan, Fan Lin, Gengming He, Zeynep Baskurt, Sangook Kim, Lin Zhang, Mohsen Esmaeili, Scott Blackman, Stephen W. Scherer, Harriet Corvol, Mitchell Drumm, Michael Knowles, Garry Cutting, Johanna M. Rommens, Lei Sun, Lisa J. Strug

**Affiliations:** 1 Program in Genetics and Genome Biology, The Hospital for Sick Children, Toronto, ON Canada; 2 The Centre for Applied Genomics, The Hospital for Sick Children, Toronto, ON Canada; 3 Program in Physiology and Experimental Medicine, The Hospital for Sick Children, Toronto, ON Canada; 4 Biostatistics Division, Dalla Lana School of Public Health, University of Toronto, Toronto, ON Canada; 5 Department of Statistics, University of Toronto, Toronto, ON Canada; 6 Department of Pediatrics, Johns Hopkins University School of Medicine, Baltimore, MA USA; 7 McLaughlin Centre and Department of Molecular Genetics, University of Toronto, Toronto, ON Canada; 8 Pediatric Pulmonary Department, Hospital Trousseau, Assistance Publique-Hôpitaux de Paris (AP-HP), Paris, France; 9 Sorbonne Universités, UPMC Univ Paris 06, INSERM, Centre de Recherche Saint-Antoine (CRSA), Paris, France; 10 Department of Pediatrics, Case Western Reserve University, Cleveland, OH USA; 11 Department of Genetics, Case Western Reserve University, Cleveland, OH USA; 12 Cystic Fibrosis Pulmonary Research and Treatment Center, University of North Carolina, Chapel Hill, NC USA; 13 McKusick-Nathans Institute of Genetic Medicine, Johns Hopkins University School of Medicine, Baltimore, MD USA; 14 Department of Molecular Genetics, University of Toronto, Toronto, ON Canada

## Abstract

Does genotype imputation with public reference panels identify variants contributing to disease? Genotype imputation using the 1000 Genomes Project (1KG; 2504 individuals) displayed poor coverage at the causal cystic fibrosis (CF) transmembrane conductance regulator (*CFTR*) locus for the International CF Gene Modifier Consortium. Imputation with the larger Haplotype Reference Consortium (HRC; 32,470 individuals) displayed improved coverage but low sensitivity of variants clinically relevant for CF. A hybrid reference that combined whole genome sequencing (WGS) from 101 CF individuals with the 1KG imputed a greater number of single-nucleotide variants (SNVs) that would be analyzed in a genetic association study (*r*^2^ ≥ 0.3 and MAF ≥ 0.5%) than imputation with the HRC, while the HRC excelled in the lower frequency spectrum. Using the 1KG or HRC as reference panels missed the most common CF-causing variants or displayed low imputation accuracy. Designs that incorporate population-specific WGS can improve imputation accuracy at disease-specific loci, while imputation using public data sets can omit disease-relevant genotypes.

## Introduction

Genotype imputation enables the integration of genome-wide data for consortia research, improves genotype density for fine-mapping, and can result in considerable cost-savings. But does this translate into imputation of disease-relevant haplotypes? Cystic fibrosis (CF) (MIM: 219700) is caused by dysfunction of the CF transmembrane conductance regulator (*CFTR* [MIM: 602421]). *CFTR* displays substantial allelic heterogeneity with greater than 300 variants reported to be disease-causing^1^ with some relatively frequent (e.g. the p.Phe508del 3 bp in-frame deletion; 70% of European CF chromosomes) but most spanning the rarer frequency spectrum (e.g. p.Gly551Asp; 2.11% of CF chromosomes according to the Clinical and Functional Translation of CFTR database or CFTR2; https://www.cftr2.org).^2^ Therefore, *CFTR* can illustrate what is gained and missed from imputation on a well-studied disease-specific locus to evaluate the relative merits of different imputation designs.

## Results

### Motivation

We initially used the 1000 Genomes (1KG; 2504 individuals)^3^ as reference to impute chromosome 7 for 1995 CF participants from the International CF Gene Modifier Consortium.^4^ The most common CF-causing allele p.Phe508del (g.117199646-117199648delCTT; hg19) was imputed with good accuracy (*r*^2^ = 0.77; *r*^2^ is a predicted correlation measure between the imputed and true genotypes). However, all other CF-relevant variants with allele frequencies >1% in CF populations based on the CFTR2 database,^2^ such as p.Gly551Asp (g.117227860 G > A), p.Trp1282Ter (g.117282620 G > A), p.Gly542Ter (g.117227832 G > T), p.Asn1303Lys (g.117292931 C > G), and p.Arg117His (g.117171029 G > A) were not present in the 1KG reference panel; consequently they were not imputed and their corresponding CF-relevant haplotypes would be excluded in the genetic association analysis.

Failure to impute population-specific genomic variation has previously been highlighted,^5,6^ and the use of study-specific reference panels has recently been shown to improve imputation accuracy.^7,8^ We considered imputation of chromosome 7 using two alternative reference panels: (1) the Haplotype Reference Consortium (HRC)^9^ resource of 32,470 individuals, to improve the probability of having as many population haplotypes as possible including CF causing haplotypes; and (2) a hybrid reference that combines the 2504 individuals from the 1KG with 101 Canadian individuals with CF sequenced at high read depth (30×), to provide enrichment with disease-specific haplotypes. The 101 Canadians with CF chosen for sequencing had severe *CFTR* genotypes that was representative of the Canadian CF population, and had comprehensive clinical data spanning several decades.

**Fig. 1:**
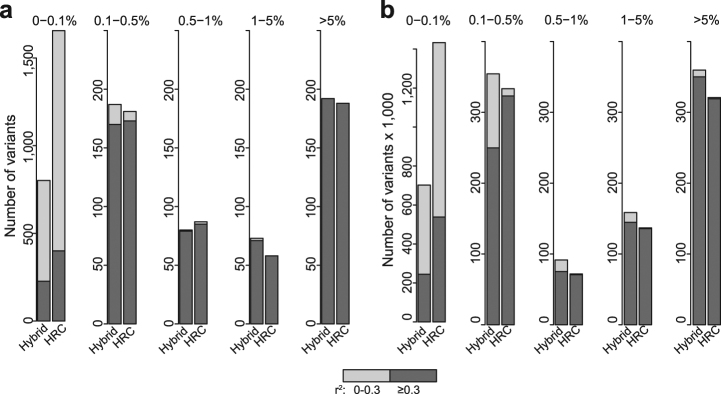
Comparison of the number of variants imputed and predicted imputation accuracy stratified by minor allele frequency using the hybrid vs. HRC reference panels. The number of single-nucleotide variants or SNVs (indels were excluded from the hybrid for a fair comparison with HRC) in **a** the *CFTR* region (chr7:117,110,017–117,318,718; hg19) and **b** chromosome 7 minus the *CFTR* region. Multi-allelic variants are counted as one occurrence. *r*^2^ is the predicted imputation accuracy. The hybrid reference improves the number of SNVs imputed over the HRC for both the *CFTR* region and the full chromosome 7 for variants with MAF ≥ 1%

Construction of the hybrid reference panel is outlined in Supplementary Fig. 1 with 1,805,357 single-nucleotide variants (SNVs) on chromosome 7. The quality control procedures for the HRC and hybrid imputation approaches were aligned to the best of our knowledge.^9^ Figure 1 displays the number of imputed variants and predicted *r*^2^ that result from imputation of the 1995 individuals with CF genotyped on the Human660W-Quad BeadChip using the two different reference panels.

### Imputation with the HRC reference panel

Submission of our data to the Michigan Imputation server^10^ which uses the HRC (r1.1)^9^ yielded 2158 SNPs in the *CFTR* region, from which 901 biallelic SNPs indicated a minimac^10^ quality score *r*^2^ ≥ 0.3. The vast majority of these 2158 variants, however, were rare with only 333 having an in-sample MAF ≥ 0.5% (Table 1). Imputation of indels by the HRC is not currently supported due to the poor call rate of this type of variation with low read-depth sequencing.^9^ Therefore the most common CF-causing variant p.Phe508del, was not imputed. Figure 1 demonstrates that the different imputation panels are advantageous for different allele frequency ranges, with the main advantage of HRC being the ability to impute a large number of general population rare variants (MAF < 0.5%) with reasonable mean predicted *r*^2^ (Fig. 1).

**Table 1:** Number of variants across the two imputation study designs

	Full chromosome 7/chr7:117,110,017-117,318,718^a^			
Method	Total^b^ (no *r*^*2*^ or MAF filters)	*r*^*2*^ ≥ 0.3^c^	MAF ≥ 0.5%	Num. SNVs^d^
Hybrid (*n* = 2605)	1,805,357/1474	1,162,772/819	681,396/397	570,436/342
HRC (*n* = 32,470)	2,283,806/2158	1,375,928/901	529,637/333	525,662/331

*MAF* minor allele frequency, *SNV* single-nucleotide variant

Positions with multiple alleles were counted as one variant

^a^Coordinates for the CFTR region with 10 kb up-stream and down-stream (hg19)

^b^Total column includes small indels while the other columns do not

^c^*r*^2^ is the minimac *r*^2^ provided by Minimac3^10^

^d^SNVs with *r*^2^ ≥ 0.3 and MAF ≥ 0.5%

### Imputation with the hybrid reference panel

Excluding indels, the hybrid reference resulted in the greatest number of imputed SNVs with *r*^2^ ≥ 0.3 and MAF ≥ 0.5% across chromosome 7 (typical parameters used for SNP inclusion in genetic association analyses). The hybrid imputation contained 1474 variants in the *CFTR* region, and enabled the imputation of 819 variants with *r*^2^ ≥ 0.3, and 397 with MAF ≥ 0.5% (Table 1). Comparing the predicted *r*^2^ for the full chromosome to the *CFTR* region (Fig. 1) indicated that the gain in accuracy from the hybrid reference is most significant in the *CFTR* region. The gain in mean predicted *r*^2^ was similar to the imputation with HRC in the *CFTR* region for the rare variants (MAF < 0.5%) and significantly improved for variants with MAF ≥ 0.5% (Fig. 1).

### Assessing the sensitivity and specificity of imputation

Motivated by the observation that imputation with the 1KG alone could not impute CF-relevant variants, we compared the imputation results from the hybrid and HRC for several known CF-causing variants with frequencies above 1% in the CF patient population, p.Gly551Asp, p.Trp1282Ter, p.Gly542Ter, p.Asn1303Lys, p.Arg117His. We used the CF-causing alleles provided in patient medical records to calculate the sensitivity. Variants successfully imputed using the hybrid reference displayed greater sensitivity (Table 2). The hybrid enabled accurate imputation of the most common p.Phe508del variant: with sensitivity and specificity of 98.5% and 88.2%, respectively. Although the HRC could not impute p.Phe508del, the HRC imputed other SNVs of interest but did so with poor sensitivity, despite reporting a relatively high imputation quality score (Table 2). Meanwhile the hybrid reference, which augments the 1KG reference with only 101 CF samples, enabled imputation with good sensitivity for the majority of mutations considered, accompanied by high imputation quality scores. It should be noted that p.Asn1303Lys could not be imputed accurately by either reference panel, and the p.Arg117His variant was absent from the hybrid reference but was successfully imputed by HRC due to its presumed presence in the much larger HRC reference panel (Table 2), highlighting the limitation of a small disease-specific reference panel.

**Table 2:** Comparison of sensitivity and specificity of *CFTR* variants imputed using hybrid vs. HRC reference panel

					Sensitivity(%), Specificity(%)	
*CFTR* variant^a^	Frequency in European HRC^b^/1KG	Frequency in CF^c^	Number of alleles in 101^d^ clinical records	Number of alleles in 1995 clinical records	HRC reference (Minimac *r*^*2*^)	Hybrid reference (Minimac *r*^*2*^)
rs113993960 (p.Phe508del)	NA / 0.40%	69.86%	159	2255	–	98.5, 88.2 (0.914)
rs75527207 (p.Gly551Asp)	0.028% / NA	2.11%	2	106	70.8, 99.93 (0.892)	94.3, 99.7 (0.927)
rs77010898 (p.Trp1282Ter)	0.010% / NA	1.22%	6	60	28.3, 100 (0.730)	96.7, 99.8 (0.964)
rs113993959 (p.Gly542Ter)	0.025% / NA	2.54%	7	116	20.7, 100 (0.738)	70.7, 99.93 (0.874)
rs80034486 (p.Asn1303Lys)	NA / NA	1.57%	2	48	–	4.2, 100 (0.380)
rs78655421 (p.Arg117His)	0.224% / NA	1.31%	0	18	100, 99.97 (0.929)	–

^a^The *CFTR* variants chosen for comparison are reported as having a frequency of at least 1% in the Clinical and Functional Translation of CFTR database (CFTR2; https://www.cftr2.org)

^b^HRC reference excluding the 1KG

^c^Allele frequencies as reported in the CFTR2 database

^d^101 individuals with CF incorporated with the 2504 from the 1KG to generate the hybrid reference panel

## Discussion

Using the *CFTR* locus as proof-of-concept, we demonstrate the extent to which reference panels that incorporate disease-specific haplotypes can improve imputation of disease-relevant variants, even with the addition of only 101 individuals with disease. The most common CF-causing p.Phe508del variant was missing from the HRC. The imputation of other CF-relevant variants with allele frequencies of 1-3% in the CF population, variants that would in general be analyzed in genetic association studies, displayed low sensitivity (but in some cases high imputation quality score) despite using an imputation reference panel with 32,470 individuals.

As reference populations grow in size, imputation quality and coverage should improve (presuming support for imputation of indels improves). However, one does not know in general whether a reference panel has adequate representation for the given disease under study. The incorporation of high read-depth in-sample WGS with public reference sequence is advantageous when possible as it ensures the existence of disease-specific haplotypes that can be imputed with good accuracy. Here we used high read-depth WGS from 101 individuals with CF to generate the hybrid reference. Of course, the greater the number of patients on whom in-sample high read depth WGS is available to generate the hybrid reference, the better the imputation will be in the most disease-relevant regions.

In summary, imputation with reference panels that incorporate disease-specific haplotypes improve sensitivity and coverage of variants in disease-relevant regions, which may or may not be known. Constructing hybrid reference panels with in-sample high-coverage WGS, when possible, is a more advantageous study design as it enhances the ability to impute disease-relevant haplotypes that will translate into improved fine-mapping and causal-variant identification.

## Methods

### Sample collection

DNA samples from 2012 individuals with CF from the North American CF Gene Modifier consortium comprised of individuals from the Canadian CF Gene Modifier Study, The University of North Carolina/Case Western Reserve Gene Modifier Study and The Johns Hopkins University Twin and Sibling Study. The research ethics boards at each collaborating institution approved the study and all patients gave signed consent to participate in the study.

### Genotyping and quality control

All 2012 individuals with CF were genotyped on the Illumina Human660W-Quad BeadChip, which consists of 655,214 SNPs. After quality control (removal of SNPs with call rate <90%, heterozygous haploid SNPs or SNPs in duplicate positions, and samples with high heterozygosity rate or mismatched for reported and genotyped sex), a total of 557,520 SNPs and 1995 individuals remained for imputation. Samples were ensured to cluster with the Utah residents with Northern and Western European ancestry from the CEPH collection (CEU) and Toscani in Italia (TSI) in the International Hapmap 3 Project^11^ through principal component analysis. Finally, all chromosome 7 SNPs were extracted prior to strand alignment (30,163 SNPs).

### Complete genomics (CG) whole-genome sequencing (WGS)

We selected 101 CF patients for WGS at Complete Genomics Inc. (CG) for mean depth coverage of 30×. These 101 individuals were chosen to have a severe *CFTR* genotype distribution that was representative of the Canadian CF population, and to have comprehensive clinical data spanning several decades. These patients were sequenced as part of our ongoing genetic studies of CF. Quality control steps per sample and per SNP were performed by CG’s proprietary pipeline. CGA tools v1.8.0.1 was used to assemble all sequenced files into variant call format (VCF). At this step, individual variants marked as low quality or without the “PASS” filter were set to missing. Monomorphic, and singleton variants were removed. Structural variants were not incorporated into the VCF file. The combined CG WGS VCF file of 101 individuals with CF contained 1,654,299 variants on chromosome 7. For duplicate alleles (same position, reference and alternate alleles), we kept the record with the lowest missing rate. Multi-allelic variants were split into bi-allelic records, followed by the removal of variants with a missing rate >10%. After QC, and prior to merging with the 1KG reference panel, the chromosome 7 WGS VCF file contained 510,399 variant records.

### Constructing the hybrid reference panel

We constructed a hybrid reference panel consisting of data from 2504 individuals from the 1000 Genomes project^3^ (1KG) and WGS from 101 Canadians with CF. This hybrid reference panel used the Phase 3 haplotype data from the 1KG as described in http://bochet.gcc.biostat.washington.edu/beagle/1000_Genomes_phase3_v5a/READ_ME_beagle_ref. Briefly, the variants removed from the 1KG were those with minor allele count less than 5, structural variants, and duplicate variants. We took the union of markers from the two data sets (for details, see flowchart provided in Supplementary Fig. 1) with 1,805,357 variants on chromosome 7, an increase of 5837 variants compared to the 1KG alone. Matching variants are merged with the 1KG, but among the multi-allelic variants, variants unique to the 1KG only were removed.

### Strand alignment of genotype data for the hybrid reference

The Beagle strand alignment utility conform-gt version 24May16.cee.jar was used with the European subset of the 1KG phase 3 (503 individuals) to align the (QC-filtered) chromosome 7 genotype data to the plus strand. It is important for this step to use the European subset of the 1KG to match with the ethnic background of the target sample as conform-gt determines strand alignment of the target sample by frequency and correlation tests with the reference. For the hybrid reference strand alignment, the WGS from the 101 Canadians with CF and the 503 European 1KG were used as reference. Strand alignment with the hybrid reference removed a total of 87 variants across chromosome 7 (81 due to absence of the variants in the reference and 6 due to inability to align ambiguous SNPs).

### Phasing and imputation using the hybrid reference

Eagle (version 2.3.2) was used for phasing and Minimac3 (version 2.0.1) for imputation of chromosome 7 with the hybrid reference panel using the full 1KG reference panel of 2504 individuals plus WGS from 101 individuals with CF. We ran the default parameters for phasing/imputation with Eagle/Minimac3, which is the same method implemented by the Michigan Imputation Server^10^ as outlined in https://imputationserver.sph.umich.edu/start.html#!pages/pipeline (accessed Feb. 5th, 2018).

### Phasing and imputation using the HRC

The chromosome 7 VCF file generated after genotyping quality control was strand-aligned using the suggested HRC-1000G-check-bim.pl script with the –n option to turn off the removal of variants due to MAF differences between the reference panel and the CF genotyped sample. For the full chromosome 7, only 89 variants were removed due to no matches found in the HRC reference, and this file was submitted to the Michigan Imputation Server^10^ (https://imputationserver.sph.umich.edu; submission date December 20th, 2017) for imputation using the HRC r1.1^9^ reference panel.

### Code availability

In-house scripts were used to construct the hybrid reference, and these can be made available upon request to the corresponding author LJS.

### Data availability statement

Information on the allele frequencies in the patient population with cystic fibrosis can be accessed at https://cftr2.org. Variant call format (VCF) files used for the 1KG (phase 3) sequencing project are available at http://bochet.gcc.biostat.washington.edu/beagle/1000_Genomes_phase3_v5a/. The HRC’s allele frequencies used for the strand alignment step can be downloaded from http://www.haplotype-reference-consortium.org/home, while the strand alignment tools can be accessed at https://faculty.washington.edu/browning/conform-gt.html (conform-gt) and http://www.well.ox.ac.uk/~wrayner/tools/ (HRC-1000G-check-bim.pl). CGA tools, used for the creation of the VCF file from the 101 sequenced patients with CF, is accessible at cgatools.sourceforge.net. Patient informed consent dictates that the genetic data be made available only for cystic fibrosis research.

## Electronic supplementary material

Supplementary Figure 1(DOCX 1547 kb)
