# Supplementary material for: Improving imputation in disease-relevant regions: lessons from cystic fibrosis
Source: NPJ Genom Med. 2018 Mar 20;3:8. doi: 10.1038/s41525-018-0047-6 (PMC5861096; doi:10.1038/s41525-018-0047-6)
Supplement: Supplementary file 1 — Supplementary Figure 1(DOCX 1547 kb) [file 41525_2018_47_MOESM1_ESM.docx]

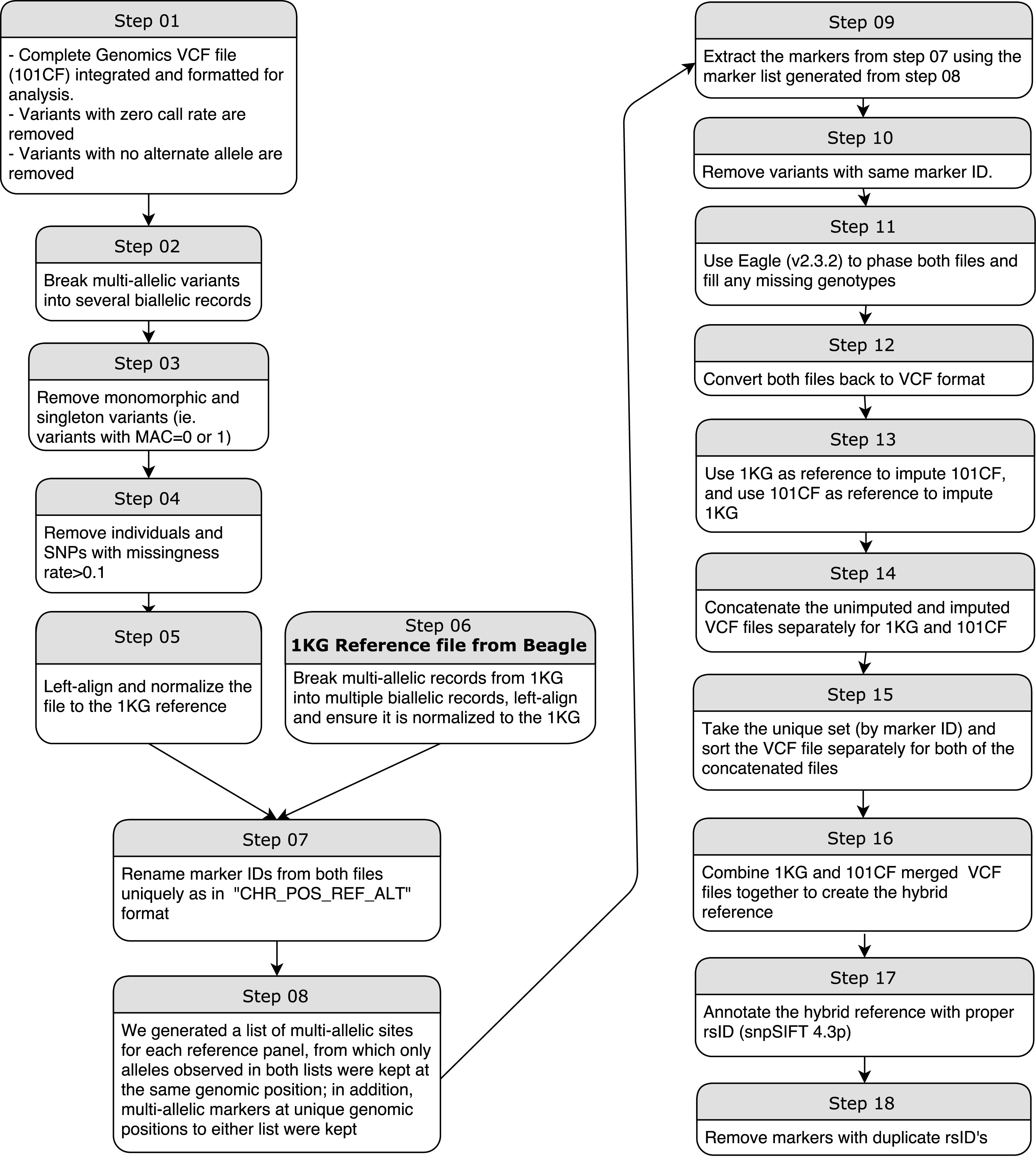


Supplementary Figure 1. Flowchart of steps followed to generate the hybrid reference panel (1KG reference panel with 101 whole genome sequenced individuals with Cystic Fibrosis).
